# A new *Muricea* species (Cnidaria, Anthozoa, Octocorallia) from the eastern tropical Pacific

**DOI:** 10.3897/zookeys.629.10828

**Published:** 2016-11-07

**Authors:** Odalisca Breedy, Hector M. Guzman

**Affiliations:** 1 Centro de Investigación en Estructuras Microscópicas, Centro de Investigación en Ciencias del Mar y Limnología, Escuela de Biología, Universidad de Costa Rica. P.O. Box 11501-2060, Universidad de Costa Rica, San José, Costa Rica; 2 Smithsonian Tropical Research Institute, P.O. Box 0843-03092, Panama, Republic of Panama

**Keywords:** Alcyonacea, Cnidaria, eastern Pacific, mesophotic zone, Muricea subtilis, new species, plexaurid, soft corals, taxonomy

## Abstract

The genus *Muricea* is considered abundant and widely distributed along the eastern Pacific. Its occurrence in shallow waters has been recognised; however species from deeper than 30 m have been rarely recorded. During the 2005 R/V Urracá expedition along the north and central Pacific coast of Costa Rica several octocoral specimens were collected by bottom trawling from 30 to 150 m yielding new species and new records. Herein we describe a new species of *Muricea* from deeper than 30 m. The morphological characters of the species were analysed and illustrated by optic and scanning electron microscopy. *Muricea subtilis* **sp. n.** can be distinguished from the other species in the genus by its thin spiny branches, non-imbricate calyces, white colony and sclerites, and the size and composition of sclerites. Comparative character tables are provided for the closest *Muricea* species-group. This new species increases the number in the genus to 26, and contributes to the knowledge on the diversity and distribution of mesophotic soft corals in the eastern Pacific.

## Introduction

The genus *Muricea* is considered abundant and widely distributed in shallow waters (< 30 m) along the eastern Pacific and was recently revised and updated to contain 25 valid species (Breedy and Guzman 2015, 2016). *Muricea* has been reported from Cape Hatteras, North Carolina to Brazil, including Bahamas, Greater and Lesser Antilles, and Caribbean islands (Bayer 1961); it also occurs in the eastern Pacific from southern California to Perú and presumably in Chile (Breedy and Guzman 2016).

*Muricea midas* Bayer, 1959 is the deepest record for the genus, at 201 m in the western Atlantic (Bayer 1959); and *Muricea fruticosa* Verrill, 1869, is known to 102 m in the eastern Pacific. *Muricea galapagensis* Deichmann, 1936, known from 94 m, was only once collected. Normally, the genus occurs shallower from one meter in intertidal zones to 30 m deep (Breedy and Guzman 2016). However, several species have been found in deeper mesophotic zones requiring further exploration and taxonomic work.

According to Breedy and Guzman (2016) boundaries among species of *Muricea* (as in many other octocorals) are difficult to draw. However, the morphological characters such as colony and sclerite shapes, sizes and colours still represent a valid approach to determine species together with field observation (e.g. habitat, bathymetry). The genus was divided in four groups according to the morphology of colonies and sclerites: the *Muricea squarrosa* species-group, *Muricea fruticosa* species-group, the *Muricea austera* species-group and the *Muricea plantaginea* species-group (Breedy and Guzman 2015, 2016).

Herein we describe a new mesophotic *Muricea* species collected during the 2005 R/V Urracá-STRI expedition to the Pacific coast of Costa Rica, that resulted in interesting material from deeper waters (see Vargas-Castillo 2008).

## Material and methods

The specimens were collected by bottom trawling from unexplored habitats down to 70 m deep in the middle mesophotic zone (from 40 to 150 m), on board of the Smithsonian Tropical Research Institute R/V Urracá along the north and central Pacific coast, from Santa Elena Bay to the Nicoya Gulf.

The specimens were fixed in 70% ethanol or air-dried. For microscopic study, they were prepared according to the protocol described by Breedy and Guzman (2002), and observed using optic microscopy, Olympus LX 51 inverted microscope, and scanning electron microscopy, with a Hitachi 3700 at the Research Center of Microscopic Structures (CIEMIC) of the University of Costa Rica (UCR) and a Zeiss EVO 40 at the Electron Microscopy Laboratory (Tupper Research and Conference Center). The holotype and paratypes are deposited in the Museo de Zoología, Universidad de Costa Rica (MZUCR).

The taxonomic approach was by the evaluation of characters following Breedy and Guzman (2015, 2016). Morphological characters of colonies and sclerites are presented in Tables 1–2 and comparison with the type material of the related taxa in the genus. Measurements of branches are given taking in account the length of the calyces whether preserved in ethanol or dry. Terminology used in descriptions mostly follows Bayer et al. (1983) and Breedy and Guzman (2015, 2016).

**Table 1.** Diagnostic characters of sclerites in the *Muricea plantaginea* species-group. Measurements given are from the holotypes and lectotypes, in mm.

Species	Sclerite colours	Anthocodial sclerite colours	Dominant type of coenenchymal and calycular sclerites	Coenenchymal and calycular spindles maximum size	Anthocodial maximum size
*Muricea plantaginea*	rb, amb/w	lo, lb/w	ls	1×0.2	0.25×0.08
*Muricea californica*	ro, ly, amb	lo	ls	0.54×0.2	0.23×0.06
*Muricea mortensenii*	w	w	s	0.7×0.12	0.21×0.08
*Muricea subtilis* sp. n.	w	w	ls	0.93×0.14	0.20×0.05

Colours: amb , amber ; lb , light brown ; lo , light orange ; rb , reddish brown ; ro , reddish orange ; w , white, colourless . Type of coenenchymal and calycular sclerites: ls , leaf-like spindle ; s , spindles .

**Table 2.** Diagnostic characters of colony morphology in the *Muricea plantaginea* species-group. Measurements given are from holotypes and lectotypes, in mm.

Species	Colony colour	Colony shape	Branching pattern	Length of unbranched terminal branchlets	Diameter of end branchlets (mm)	Calyx height at branchlets	Calyx arrangement at branchlets
*Muricea plantaginea*	db/w	fla	irr, lat	10–50	2–3	0.7–1.2	c, imbr
*Muricea californica*	ro	bu	irr, lat	0.5–2.8	3–3.2	1.1–1.9	c, slightly imbr
*Muricea mortensenii*	py	fla	irr	2–4	2–3	0.7–1	c
*Muricea subtilis* sp. n.	py,w	lat, fla	irr, lat, dich	5–40	1.5–2	1–1.2	c

Colours: db , deep brown ; py , pale yellow ; ro , reddish orange ; w , white, colourless . Colony shape: bu , bushy ; fla , fan-like, flabelliform . Branching pattern: dich , irregularly dichotomous ; irr , irregular ; lat , lateral . Calyx arrangement at branchlets: c , close, not imbricate ; imbr , imbricate .

## Results

### Class Anthozoa Ehrenberg, 1834 Subclass Octocorallia Haeckel, 1866 Order Alcyonacea Lamouroux, 1812 Family Plexauridae Gray, 1859

#### Genus Muricea

Taxon classificationAnimaliaAlcyonaceaPlexauridae

Lamouroux, 1821

Muricea Lamouroux, (pars.) 1821: 36; Blainville (pars) 1834: 509; Ehrenberg (pars.) 1834: 134; Dana 1846: 673; Milne Edwards and Haime 1857: 142; Kölliker 1865: 135; Verrill 1868: 411; Verrill 1869: 418–419, 450; Studer 1887: 58; Wright and Studer 1889: 93; Gorzawsky 1908: 8; Nutting 1910: 9; Kükenthal 1919: 835; 1924: 141; Riess 1929: 383–384; Aurivillius 1931: 102–104; Deichmann 1936: 99; Bayer 1956: F210; 1959: 12; 1961: 179–180; 1981: 930 (in key); 1994: 23–24; Tixier-Durivault 1970: 154; Harden 1979: 140; Hardee and Wicksten 1996: 127–128; Marques and Castro 1995: 162; Castro et al. 2010: 779; Breedy and Guzman 2015: 6–7; 2016: 7–9.
Eumuricea (pars.) Verrill, 1869: 449; Riess 1929: 397; Breedy and Guzman 2015: 6–7.

##### Type species.

*Muricea spicifera* Lamouroux, 1821, by subsequent designation (Milne Edwards and Haime 1857.)

##### Genus diagnosis

(based on Breedy and Guzman 2016). Colonies planar or multiplanar, bushy, arborescent, laterally branched, pinnately branched, dichotomous or with long flexible branches, with some occasional branch anastomosis. Branches and branchlets upward bending almost parallel, and with about the same thickness all along, frequently with slightly enlarged tips. Coenenchyme moderately to very thick (compared to other plexaurids) with a circle of longitudinal canals surrounding the axis and dividing the coenenchyme into a thin inner layer or axial sheath, and a thicker outer layer. The outer and inner layer of coenenchyme indiscriminate, almost blended in species with thinner branches. In some species with a thin coenenchyme polyps fully retractile within prominent calyces longitudinally and closely placed all around branches and branchlets, or spaced in loose spirals around branches and branchlets. Calyces prominent, shelf-like or tubular, with prickly projecting spindles, longitudinally arranged. Base of anthocodia without sclerites or with flat rods arranged in weakly differentiated collaret and points below tentacles, or just transversely set along the neck zone of polyp. Sclerites of outer coenenchyme and of calyx mostly long, unilateral spinous spindles, often massive, sculptured on inner surface by crowded complex tubercles and on outer surface by simple spines or prickles, and in some species with a few more or less prominent coarse, prickly projections. Spindles with laterally placed spinous or leaf-like processes are the dominant type in some species. Axial sheath composed of capstans, spindles, or oval forms, and undeveloped sclerites. Sclerite colours are white, various hues of yellow, amber, orange, purple and red. Anthocodials with lower hues.

#### Muricea subtilis sp. n.

Taxon classificationAnimaliaAlcyonaceaPlexauridae

http://zoobank.org/23F8B95D-10AB-4AC2-A4FC-EF1326AD0765

Figures 1 , 2 , 3

##### Material.

Holotype: UCR 2322 (URR 46), ethanol preserved, off Esterillos, Puntarenas, Central Pacific, Costa Rica, 09°20.940'N, 84°30.240'W–09°21.242'N, 84°30.043'W, 51.7–53 m, R. Vargas, R/V Urraca, 17 July 2016. UCR 2322A, fragment for molecular analysis in progress.

Paratypes: MZUCR-OCT 0082 (URR 44), ethanol preserved, off Punta Mala, Puntarenas, 09°22.085'N, 84°32.206'W–09°22.280'N, 84°32.037'W, 44.2–44 m, R. Vargas, 17 July 2005; MZUCR-OCT 0125 (URR 26–53), dry, off Carrillo Beach, Nicoya, Guanacaste, 09°51.264'N, 85°29.37'W–09°50.727'N, 85°29.37'W, 39–40 m, R. Vargas, R/V Urraca, 16 July 2005; MZUCR 0126 (TWL 27–36), dry, off Carrillo Beach, 09°50.013'N, 85°29.476'W–09°49.88'N, 85°29.40'W, 30–32 m, R. Vargas, R/V Urraca, 16 July 2005; MZUCR 0140 (URR 47), dry, off Esterillos, 09°20.212'N, 84°28.358'W–09°21.610'N, 84°28.275'W, 51.7–53 m, R. Vargas, R/V Urraca, 17 July 2016; UCR 2321 (URR 46), as the holotype.

##### Type locality.

09°20.940'N, 84°30.240'W (off Esterillos, Puntarenas), 53 m in depth.

##### Diagnosis.

Colonies spiny and delicate in appearance, fan-like or lateral. Branching irregular, mostly dichotomous, in one or two planes. Branches and branchlets thin, 1.5–2 mm in diameter, in some cases thinner, about 1 mm. Some branch pseudo-anastomosis present. Polyps mostly close together. Calyces shelf-like, prominent, up to 1.2 mm. Calyces not imbricate. Coenenchyme thin. Coenenchymal and calycular sclerites mostly leaf-like spindles up to 0.95 mm long. Anthocodial sclerites mostly irregular warty rods and thin torches, translucent or whitish. Colony colour whitish to pale yellow.

##### Description.

**Figure 1.**
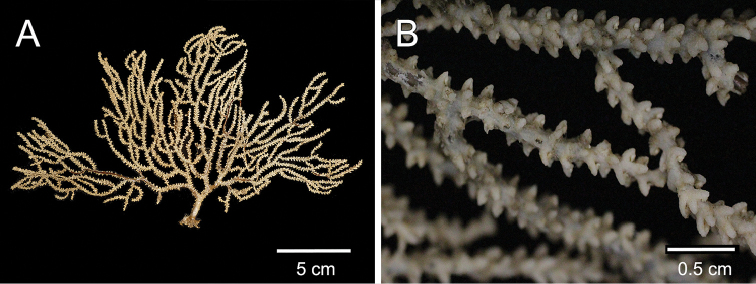
*Muricea subtilis* sp. n., UCR 2322 (holotype). **A** Colony **B** Detail of branches.

**Figure 2.**
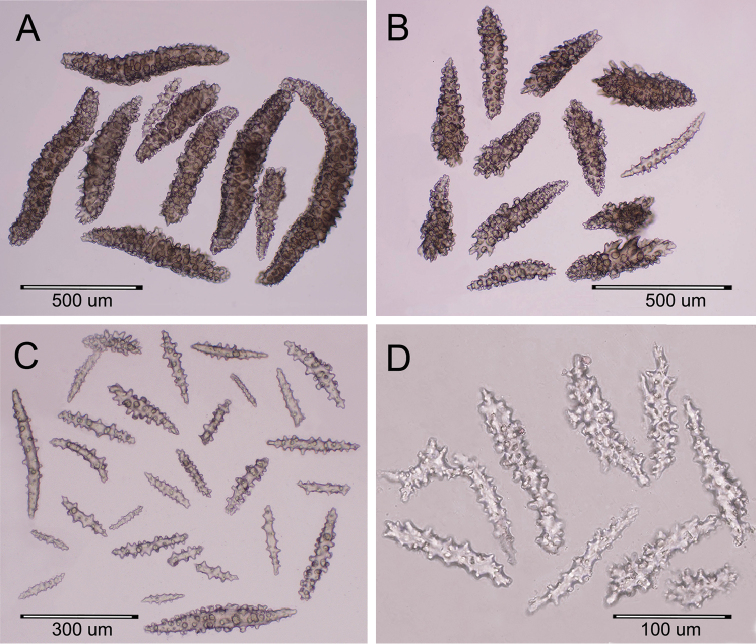
*Muricea subtilis* sp. n., UCR 2322 (holotype). **A–C** Coenenchymal sclerites **D** Anthocodial sclerites (optic micrographs).

**Figure 3.**
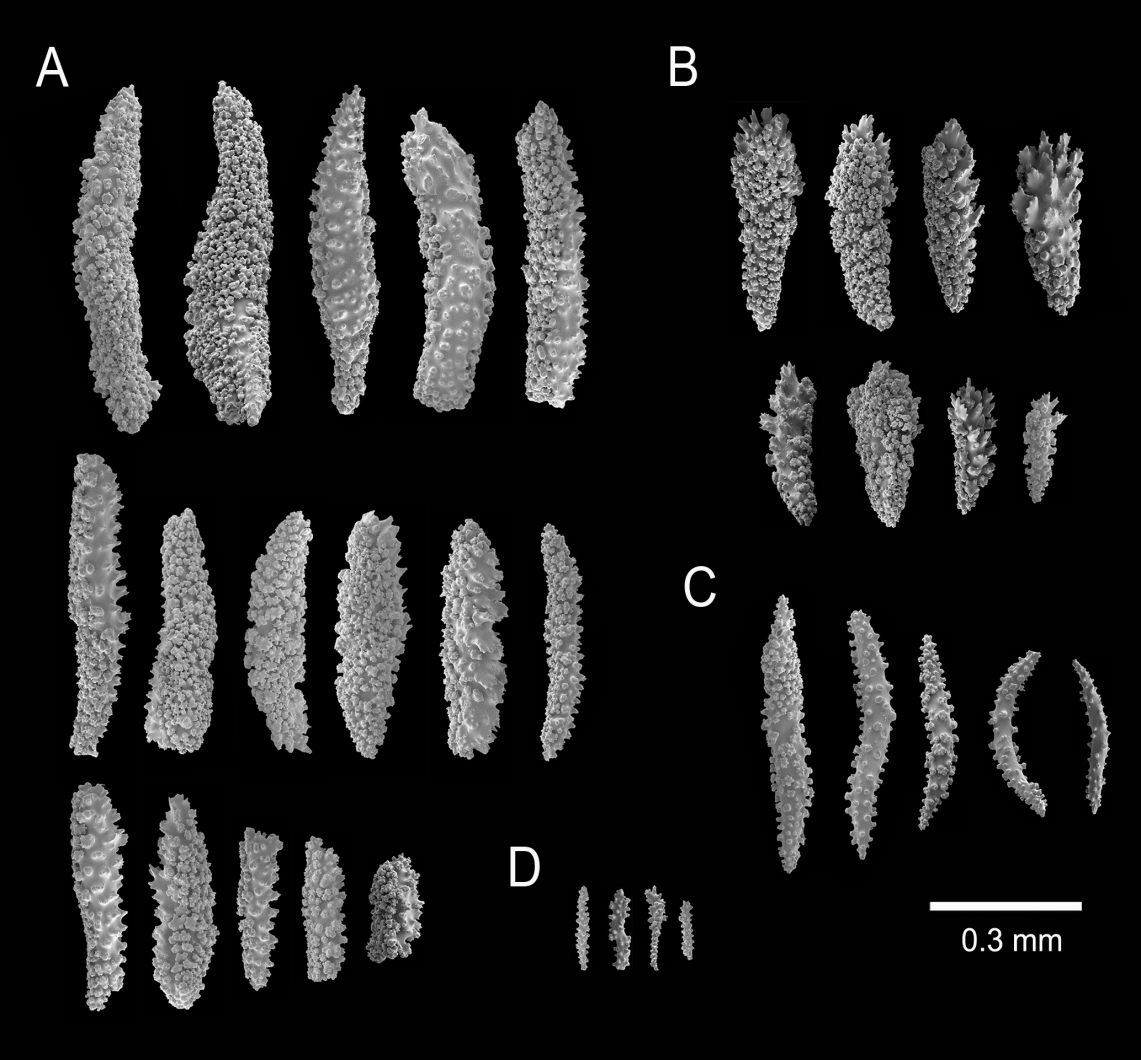
*Muricea subtilis* sp. n., UCR 2322. **A–B** Calycular and coenenchymal sclerites **C** Axial sheath **D** Anthocodial sclerites.

The holotype is a 14.5 cm tall and 23 cm wide colony. A 15 mm long stem, 6 mm in diameter, subdivide in two main branches, 4–5 mm diameter and arise from an irregular, 15 mm diameter holdfast (Figure 1A). The branches are about the same diameter at the bottom of the colony 3–4 mm producing thinner branchlets 2–3 mm diameter up to the ends. Branching is irregular, mostly dichotomous, branches and branchlets project at angles 45°–75°and separated up to 25 mm. They spread in one plane in a fan-like colony. The branchlets are straight or curved inwards, some are anastomosed. Unbranched terminal ends are about 2 mm in diameter and up to 40 mm long. The axis is amber. The calyces are shelf-like, 1–1.2 mm long, giving a spiny appearance to the colony. They are close together, or only a few millimetres apart, 0.5–1.5 mm, and not imbricate (Figure 1B). Some branches are devoid of polyps, probably eaten by worms. Polyps are on the upper side of the elongated calyces. The calyx size and spacing vary from the larger branches to the thinner, being larger and acute, and closer placed at the branchlets and shorter, and distant at the main branches and almost absent at the stem. The coenenchyme is thin, composed of whitish and translucent sclerites, mostly of various kinds of spindles (Figure 2A–C). The coenenchyme and the calycular sclerites are mostly leaf-like spindles, 0.25–0.93 mm long, and 0.09–0.20 mm wide and spindles, 0.40–0.60 mm long and 0.06–0.10 wide (Figure 3A–C). The axial sheath is composed of spindles, 0.25–0.45 mm long and 0.04–0.07 mm wide (Figure 3D). The anthocodial sclerites are translucent irregular warty rods, thin torches, irregular short spindles, 0.05–0.2 mm long, and 0.01–0.05 mm wide (Figures 2D, 3D). The colony is whitish to pale yellow (Figure 1A–B).

The paratypes agree in all characters with the holotype; however, some colonies have thinner branchlets, about 1 mm in diameter, and the leaf-like spindles can reach 0.95 mm long.

##### Etymology.

The adjective *subtilis* (L) meaning fine, thin, delicately slender, of a cutting edge, is used here, in allusion to the thin and spiny branches characteristic of the species. The term *subtilis* in literature combines sharpness and acuteness that imply clarity which could also evoke the white colour of the colony.

##### Habitat and distribution.

The species has been collected from muddy-sand bottoms, together with other octocoral species such as *Muricea fruticosa* Verrill, 1869; *Pacifigorgia senta* Breedy & Guzman, 2003, and other invertebrates from 30 to 54 m deep. A few species of gorgonians were the dominant component of the catches; some specimens were attached to debris or shells that probably hold the colonies on the mud-sandy substrate. *Muricea subtilis* sp. n. was collected along the outer part of Nicoya Gulf and central Pacific coast of Costa Rica.

## Discussion

The species belongs to the *Muricea plantaginea* species-group together with *Muricea mortensenii* and *Muricea californica*. According to Breedy and Guzman (2016) this species-group is characterised by shelf-like calyces, thin coenenchyme, thin branches and the lack of unilateral spinous spindles (as defined for the genus). The new species’ delicate spiny colony, almost immediately separates it from the others in the group. However, it is similar to *Muricea plantaginea* (Valenciennes, 1846), white variety and *Muricea mortensenii* Hickson, 1928 in the colour of the colony and sclerites. It differs from the latter in its thicker branches, shorter calyces and smaller spindles that are the dominant type of sclerites in *Muricea mortensenii* (Tables 1–2). *Muricea plantaginea* is distinguished from *Muricea subtilis* sp. n. in having thicker non-dichotomous branches, and mostly flabellate colonies with stronger structure that is evident also in small, young colonies of *Muricea plantaginea*. The imbricate calyces and larger leaf-like spindles, up to 1 mm or slightly more (Table 1–2) in *Muricea plantaginea* are also features that differentiate these two close species.

## Supplementary Material

XML Treatment for Muricea

XML Treatment for Muricea subtilis

## References

[B1] Aurivillius M (1931) The gorgonians from Dr. Sixten Bock’s expedition to Japan and the Bonin Islands, 1914. Kungliga Svenska Vetenskapsakademiens Handlingar (ser. 3) 9: 1–337.

[B2] Bayer FM (1956) Octocorallia, Part F. Coelenterata. In: Moore RC (Ed.) Treatise on Invertebrate Paleontology. Geological Society of America and University of Kansas Press, Lawrence-Kansas, 166–231.

[B3] Bayer FM (1959) Octocorals from Surinam and the adjacent coasts of South America. Studies on the Fauna of Suriname and Other Guyanas 6: 1–43.

[B4] Bayer FM (1961) The shallow-water Octocorallia of the West Indian Region – A manual for marine biologists. In: Hummelinck W (Ed.) Studies on the Fauna of Curacao and other Caribbean Islands 12(55): 1–373.

[B5] Bayer FM (1981) Key to the genera of Octocorallia exclusive of Pennatulacea (Coelenterata: Anthozoa) with diagnoses of new taxa. Proceedings of the Biological Society of Washington 94(3): 902–947.

[B6] Bayer FM (1994) A new species of the gorgonacean genus *Muricea* (Coelenterata: Octocorallia) from the Caribbean Sea. Precious Corals and Octocoral Research 3: 23–27.

[B7] Bayer FM Grasshoff M Verseveldt J (1983) Illustrated Trilingual Glossary of Morphological and Anatomical Terms Applied to Octocorallia. E.J. Brill/Dr. W. Backhuys, Leiden-the Netherlands, 75 pp.

[B8] Blainville HMD de (1834) Manuel d’Actinologie ou de Zoophytologie. FG Levrault, Paris, 694 pp.

[B9] Breedy O Guzman HM (2002) A revision of the genus *Pacifigorgia* (Coelenterata: Octocorallia: Gorgoniidae). Proceedings of the Biological Society of Washington 115(4): 782–839.

[B10] Breedy O Guzman HM (2003) A new species of *Pacifigorgia* (Coelenterata: Octocorallia: Gorgoniidae) from Panamá. Zootaxa 128: 1–10. doi: 10.11646/zootaxa.128.1.1

[B11] Breedy O Guzman HM (2015) A revision of the genus *Muricea* Lamouroux, 1821 (Anthozoa, Octocorallia) in the eastern Pacific. Part I: *Eumuricea* Verrill, 1869 revisited. ZooKeys 537: 1–32. doi: 10.3897/zookeys.537.6025 10.3897/zookeys.537.6025 PMC4714044 26798234

[B12] Breedy O Guzman HM (2016) A revision of the genus *Muricea* Lamouroux, 1821 (Anthozoa, Octocorallia) in the eastern Pacific. Part II. ZooKeys 581: 1–69. doi: 10.3897/zookeys.581.7910 10.3897/zookeys.581.7910 PMC4857041 27199581

[B13] Castro CB Medeiros MS Loyola LL (2010) Octocorallia (Cnidaria: Anthozoa) from Brazilian reefs. Journal of Natural History 44: 763–827. doi: 10.1080/00222930903441160

[B14] Dana JD (1846) Zoophytes. United States Exploring Expedition during the years 1838, 1839, 1840, 1841, 1842, under the command of Charles Wilkes, U.S.N. Vol. 7 Lea and Blanchard, Philadelphia, 740 pp. PMC10420750 38209897

[B15] Deichmann E (1936) The Alcyonaria of the western part of the Atlantic Ocean. Memoirs of the Museum of Comparative Zoology at Harvard College, Vol. LIII Cambridge, Massachsetts, 317 pp.

[B16] Ehrenberg CG (1834) Beitrage zur physiologischen Kenntniss der Corallenthiere im allgemeinen, und besonders des rothen Meeres, nebst einem Versuche zur physiologischen Systematik derselben. Abhandlungen der Königlichen preussischen Akademie der Wissenschaften zu Berlin. Aus dem Jahre 1832. Erster Theil, 380 pp.

[B17] Gorzawsky H (1908) Die Gorgonaceenfamilien der Primnoiden und Muriceiden. Inaugural-Dissertation zur Erlangung der philosophischen Doktorwurde der hohen philosophischen Fakultat der Kongelige Universität Breslau, Buchdruckerei H. Fleischmann, Breslau.

[B18] Gray JE (1859) On the arrangement of zoophytes with pinnated tentacles. Annals and Magazine of Natural History 4(3): 439–444.

[B19] Haeckel E (1866) Generelle Morphologie der Organismen. Berlin, 1036 pp. doi: 10.1515/9783110848281

[B20] Hardee M Wicksten MK (1996) Redescription and taxonomic comparison of three eastern Pacific species of *Muricea* (Cnidaria: Anthozoa). Bulletin of the Southern California Academy of Sciences 95(3): 127–140.

[B21] Harden DG (1979) Intuitive and Numerical Classification of East Pacific Gorgonacea (Octocorallia). PhD Thesis, Illinois State University, Illinois.

[B22] Hickson SJ (1928) Papers from Dr. Th. Mortensen’s Pacific Expedition 1914–16. XLVII. The Gorgonacea of Panama Bay together with a description of one species from the Galápagos Islands and one from Trinidad. Videnskabelige Meddelelser Fra Dansk Naturhistorisk Forening 85: 325–422.

[B23] Kölliker RA von (1865) Icones histiologicae oder Atlas der vergleichenden Gewebelehre. Zweite Abtheilung. Der feinere Bau der hoheren Thiere. Erstes Heft. Die Bindesubstanz der Coelenteraten. Verlag von Wilhelm Engelmann, Leipzig, 181 pp.

[B24] Kükenthal W (1919) Gorgonaria. Wissenschaftliche Ergebnisse der deutsche Tiefsee-Expeditionen “*Valdivia*” 1898–99 13(2): 1–946.

[B25] Lamouroux JVF (1816) Histoire des polypiers coralligènes flexibles, vulgairement nommés Zoophytes. F. Poisson, Caen, 560 pp. doi: 10.5962/bhl.title.11172

[B26] Lamouroux JVF (1821) Exposition méthodique des genres de l’ordre des polypiers, avec leur description et celles des principales espèces, figurées dans 84 planches; les 63 premières appartenant a l’Histoire Naturelle des Zoophytes d’Ellis et Solander. Paris, chez Mme Veuve Agasse, Paris, 115 pp. doi: 10.5962/bhl.title.11328

[B27] Marques ACSJ Castro CB (1995) *Muricea* (Cnidaria, Octocorallia) from Brazil, with description of a new species. Bulletin of Marine Science 567(1): 161–172.

[B28] Milne Edwards H Haime J (1857) Histoire naturelle des coralliaires ou polypes proprement dits, Vol. 1 à la Libraire Encyclopédique de Roret, Paris, 326 pp.

[B29] Nutting CC (1910) The Gorgonacea of the Siboga Expedition. III The Muriceidae Siboga Expedition Monograph, 13b, 108 pp.

[B30] Riess M (1929) Die Gorgonarien Westindiens. Kapitel 8. Die Familie Muriceidae. Zoologische Jahrbuecher Systematik Supplement 16(2): 377–420.

[B31] Studer T (1887) Versuch eines Systemes der Alcyonaria. Archiv für Naturgeschichte 53(1): 1–74.

[B32] Tixier-Durivault A (1970) Octocoralliaires. Campagne de la “*Calypso*” au large des côtes atlantiques de l’Amérique du Sud (1961–1962). Annales de l’Institut Oceanographique 47: 145–169.

[B33] Valenciennes A (1855) Extrait d’une monographie de la famille des Gorgonidees de la classe des polypes. Comptes Rendus Académie des Sciences, Paris 41: 7–15. doi: 10.5962/bhl.part.28683

[B34] Vargas-Castillo R (2008) Estomatópodos y decápodos (Crustacea), de la expedición R/V Urracá-STRI (2005) en las costas del Pacífico central y norte de Costa Rica. Revista de Biología Tropical 56(4): 105–112.

[B35] Verrill AE (1868) Critical remarks on halcyonoid polyps in the museum of Yale College, with descriptions of new genera. American Journal of Science and Arts 45: 411–415.

[B36] Verrill AE (1869) Notes on Radiata in the Museum of Yale College, Number 6: Review of the corals and polyps of the West Coast of America. Transactions of the Connecticut Academy of Arts and Sciences (Second Edition) 1: 418–518.

[B37] Wright EP Studer T (1889) Report of the Alcyonaria collected by H.M.S. “Challenger” during the years 1873-91876. Challenger Reports: Zoology 31(64): 1–314.

